# Investigating the Link Between Biofilm Formation and Antibiotic Resistance in Clinical Isolates of *Acinetobacter baumannii*

**DOI:** 10.1155/ijm/1009049

**Published:** 2025-02-12

**Authors:** Kasra Javadi, Poorya Ghaemian, Mana Baziboron, Abazar Pournajaf

**Affiliations:** ^1^Department of Microbiology, Faculty of Medicine, Shahed University, Tehran, Iran; ^2^Department of Microbiology, Faculty of Medicine, Babol University of Medical Sciences, Babol, Iran; ^3^Infectious Department, Rouhani Hospital, Bobol University of Medical Science, Babol, Iran

**Keywords:** *Acinetobacter baumannii*, antimicrobial susceptibility, biofilm, oxacillinases

## Abstract

**Background:** *Acinetobacter baumannii* has become a significant problem in hospitals worldwide during the last decades. Biofilm formation is a virulence factor that may affect antibiotic resistance. This study aimed to elucidate the correlation between biofilm formation and biofilm-related and oxacillinase genes in *A*. *baumannii* clinical isolates.

**Methods:** This study was conducted on 53 *A*. *baumannii* isolates collected from hospitals affiliated with Babol University of Medical Sciences (Babol, Iran) from April to October 2023. Kirby–Bauer disc diffusion was used to determine antibacterial resistance. Biofilm formation was examined using crystal violet staining. Polymerase chain reaction was used to detect oxacillinase (*bla*_OXA−23_, *bla*_OXA−24_, *bla*_OXA−51_, and *bla*_OXA−58_) and biofilm-encoding (*bap* and *bla*_PER−1_) genes using specific primers.

**Results:** The strains showed the highest resistance to trimethoprim/sulfamethoxazole and ciprofloxacin (98.11%) and the lowest resistance to ampicillin/sulbactam (66.03%). All isolates formed biofilms. Also, 67.92%, 18.86%, and 11.32% were strong, moderate, and weak biofilm producers, respectively. The frequencies of *bla*_OXA−23_, *bla*_OXA−24_, *bla*_OXA−51_, *bap*, and *bla*_PER−1_ genes were 92.45%, 71.69%, 100%, 73.58%, and 58.49%, respectively. None of the isolates harbored *bla*_OXA−58_.

**Conclusions:** A high prevalence of antibiotic-resistant strains was found among *A*. *baumannii* clinical isolates. There was no significant correlation between the clinical sample type and biofilm formation, but a notable link was found between antimicrobial resistance and biofilm formation, except for ciprofloxacin. Oxacillinase genes were not significantly correlated with biofilm formation, but biofilm production was associated with *bap* rather than *bla*_PER−1_. Understanding the *A*. *baumannii* biofilm formation process is crucial for effective control of associated infections by targeting this mechanism.

## 1. Introduction

*Acinetobacter baumannii* (*A*. *baumannii*) has become a significant problem in hospitals worldwide during the last decades [1]. *A*. *baumannii* is responsible for severe infections that may be life-threatening, such as ventilator-associated pneumonia (VAP), meningitis, bacteremia, and urinary tract infections (UTI) [2]. The mortality rate ranges from 18.26% to 88.7%, depending on the source of infection [3]. *A*. *baumannii* is at the top of the World Health Organization's critical priority list of bacteria, representing the most severe risk to human health [4].

One of the main challenges in treating infections caused by *A*. *baumannii* is its resistance to many antibiotics (including carbapenems) commonly used to treat severe infections. This means that there are few treatment options for these infections, which could lead to high mortality rates, increased hospitalizations, and increased health care costs [5]. This bacterium uses different mechanisms to resist different antibiotic classes, including the production of antibiotic-degrading/modifying enzymes, overexpression of efflux pumps, biofilm formation, and alteration of drug targets [6]. According to recent reports, the primary mechanism responsible for carbapenem resistance in *A*. *baumannii* isolates is the production of carbapenemases, including class B metallo-β-lactamases and class D beta-lactamases (oxacillinases) [7]. The production of carbapenemases, including metallo-β-lactamases, is a critical mechanism responsible for antibiotic resistance in *A*. *baumannii* [8]. These reports complement our findings on oxacillinase genes, offering a broader perspective on how different enzymatic pathways contribute to the multidrug-resistant phenotype of this pathogen.

The adaptability and survival of *A*. *baumannii* in various environments could be attributed to the plasticity of its pan-genome and the presence of multiple virulence factors. Recent studies have explored this natural genetic reservoir, highlighting its role in bacterial pathogenicity and treatment resistance [9].

*A. baumannii* pathogenesis is characterized by many virulence factors, including porins, capsules, cell wall lipopolysaccharide, enzymes, biofilm formation, motility, and iron-acquisition systems [10]. The capacity to produce biofilm on medical equipment and devices plays a crucial role in the pathogenicity of this bacterium [11]. Biofilm formation is a complex process that utilizes many factors such as biofilm-associated protein (Bap), outer membrane protein A (OmpA), β-lactamase PER1 (*bla*_PER−1_), pili-chaperone-Osher assembly system CsuA/BABCDE, and iron uptake mechanism [12]. *Bap* is essential for the construction of both three-dimensional tower structures and water channels in biofilms. *Bap*, released via a Type I secretion system, facilitates *A*. *baumannii* biofilm formation and maturation [13]. The *bla*_PER−1_ gene is also essential for biofilm formation by *A*. *baumannii* [14]. It improves the ability of this bacterium to adhere to bronchial epithelial cells and plastic surfaces. Nevertheless, the specific mechanism by which it operates is still uncertain [15]. The *bla*_PER−1_ gene significantly impacts the existence and development of antibiotic resistance characteristics in *A*. *baumannii* strains due to its contribution to biofilm formation [16].

Therefore, providing a new perspective to better understand *A*. *baumannii* clinical isolates by exploring correlations between their survival parameters as well as their potential transmission methods from the environment to the patient could help control *A*. *baumannii* nosocomial infections. Accordingly, this study aimed to elucidate the correlation between biofilm formation ability and biofilm-related and oxacillinase genes in clinical isolates of *A*. *baumannii*.

## 2. Materials and Methods

### 2.1. Bacterial Strains

In this cross-sectional study, 53 nonduplicate *A*. *baumannii* isolates were collected from sputum, blood, wound swab, urine, and cerebrospinal fluid (CSF) samples submitted to the laboratories of hospitals affiliated with Babol University of Medical Sciences (Babol, Iran) from April to October 2023. The reference strains *A*. *baumannii* ATCC 19606 and *Escherichia coli* ATCC 25922 were used as control strains in biofilm formation assays or antibiotic susceptibility tests.

### 2.2. Identification of *A*. *baumannii*

*A.baumannii* was identified through Gram staining, motility, oxidase, catalase, citrate utilization, indole, urease, and Kligler Iron Agar (KIA) tests. Additionally, confirmation was achieved by amplifying the *16S rRNA* gene.

### 2.3. Antimicrobial Susceptibility Testing

The susceptibility of the isolates to different antibacterial agents was assessed using the Kirby–Bauer disc diffusion method on Mueller–Hinton agar (MHA) plates from Padtan Teb Co., Iran. The tested antibacterial agents, classified according to their respective families, included penicillins + β-lactamase inhibitors (ampicillin/sulbactam), carbapenems (imipenem), aminoglycosides (tobramycin and amikacin), extended-spectrum cephalosporins (ceftazidime), folate pathway inhibitors (trimethoprim/sulfamethoxazole), and fluoroquinolones (ciprofloxacin). The results were interpreted based on the guidelines recommended by the Clinical and Laboratory Standards Institute [17].

### 2.4. Biofilm Formation Assay

Biofilm formation ability was assessed using the crystal violet staining method with minor modifications as previously described [18]. In brief, the overnight culture of each strain on Luria–Bertani (LB) broth was diluted to a final optical density of 600 (OD600 = 0.1). Then in duplicate, 180 μL of tryptic soy broth (TSB) containing 1% glucose and 20 μL of bacterial suspensions were introduced into a 96-well flat-bottomed polystyrene microplate (SPL, Korea). Following a 24-h incubation period at 37°C, the plates were rinsed three times with sterile deionized water and then treated with 200 μL of 0.1% crystal violet solution for 20 min. After rinsing three times with sterile deionized water, the plates were dissolved in 200 μL of 99% methanol at room temperature for 20 min. The absorbance was measured at a wavelength of 570 nm. The cut-off optical density (ODc) was determined to be three standard deviations (SD) higher than the average optical density (OD) of the negative control (TSB alone). The classification criteria were as follows: OD ≤ ODC: nonbiofilm producers; ODC < OD ≤ 2 × ODC: weak biofilm producers; 2 × ODC < OD ≤ 4 × ODC: moderate biofilm producers; and OD > 4 × ODC: strong biofilm producers [19].

### 2.5. DNA Extraction

One microliter of the overnight bacterial culture was added to 300 μL of pure molecular biology-grade water and subjected to boiling at 100°C for 10 min in a 1.5-mL tube. Subsequently, the bacterial mixture was vigorously mixed and centrifuged at 12,800 rpm for 1 min. The supernatant was used as the DNA template in polymerase chain reaction (PCR) [20].

### 2.6. Molecular Detection of Oxacillinase and Biofilm-Related Genes

PCR was performed using specific primers to detect the presence of oxacillinase (*bla*_OXA−23_, *bla*_OXA−24_, *bla*_OXA−51_, and *bla*_OXA−58_) and biofilm-encoding (*bap* and *bla*_PER−1_) genes (Table 1). PCR products were run on a 1% agarose gel with 1 × TBE (Tris-borate-EDTA) buffer. After electrophoresis, the gel was stained with safe stain load dye (SinaClon, Iran) and visualized under ultraviolet (UV) light.

### 2.7. Statistical Analysis

Statistical analyses were conducted using the SPSS software Version 27.0. The chi-squared test was used to assess categorical data. A *p* value of < 0.05 was considered statistically significant.

## 3. Results

### 3.1. Distribution and Identification of the Isolates

In this study, 53 *A*. *baumannii* strains were isolated from various clinical specimens using conventional microbiological methods. Clinical specimens included sputum (*n* = 17, 32.07%), blood (*n* = 12, 22.64%), wound swab (*n* = 10, 18.86%), urine (*n* = 10, 18.86%), and CSF (*n* = 4, 7.54%) samples.

### 3.2. Antibiotic Susceptibility Test

The antimicrobial susceptibility profiles of 53 *A*. *baumannii* isolates against seven antimicrobial agents are shown in Table 2. The isolates showed the highest resistance to trimethoprim/sulfamethoxazole (98.11%) and ciprofloxacin (98.11%), followed by imipenem (92.45%), ceftazidime (90.56%), amikacin (88.67%), tobramycin (81.13%), and ampicillin/sulbactam (66.03%), respectively (Figure S1).

### 3.3. Biofilm Formation

Out of the 53 *A*. *baumannii* isolates evaluated, all (100%) were able to produce biofilm. Specifically, 11.32% (*n* = 6) were classified as weak biofilm producers, 18.86% (*n* = 10) as moderate biofilm producers, and 67.92% (*n* = 36) as strong biofilm producers (Figure S2).  Table 3 displays the correlation between the sample type and biofilm production. No significant correlation was observed between the clinical sample type and biofilm formation ability.  Table 4 displays the correlation between antimicrobial resistance and biofilm formation ability. A significant correlation was found between antimicrobial resistance and biofilm formation, except for ciprofloxacin resistance.

### 3.4. Oxacillinase Genes

The most and least prevalent oxacillinase genes in *A*. *baumannii* isolates were *bla*_OXA−51_ (100%, 53 of 53) and *bla*_OXA−24_ (71.69%, 38 of 53), respectively. The *bla*_OXA−58_ gene was not observed in any of the clinical isolates (Figures S3, S4, S5). The most prevalent oxacillinase genes in strong, moderate, and weak biofilm-producing isolates were *bla*_OXA−24_, *bla*_OXA−24_, and *bla*_OXA−23_, respectively (Table 5).

### 3.5. Biofilm-Encoding Genes

The biofilm-related genes in *A*. *baumannii* isolates were *bap* (73.58%, 39 of 53) and *bla*_PER−1_ (58.49%, 31 of 53) (Figure S3). The most prevalent biofilm-encoding genes in strong and moderate biofilm-producing isolates were *bap* and *bla*_PER−1_, respectively (Table 6).

## 4. Discussion

Biofilm production is considered a significant pathogenic characteristic that plays an important role in the development and spread of *A*. *baumannii* infections [24]. The present study evaluated the genes responsible for biofilm production and antibiotic resistance as well as the relationship between these genes and biofilm formation in clinical isolates of *A*. *baumannii*.

In the current investigation, *A*. *baumannii* isolates exhibited the highest resistance to trimethoprim/sulfamethoxazole and ciprofloxacin (98.11%) and the lowest resistance to ampicillin/sulbactam (66.03%). In a study by Rouhi, Safarkar, and Habibi, the highest resistance was related to ceftazidime (93.15%) and imipenem (94.52%), and the lowest resistance was related to amikacin (47.94%) [25]. In another study by Ghadiri and colleagues (2023), the highest resistance was related to ciprofloxacin and imipenem (both at 100%), while the lowest resistance was related to colistin (100%) [26]. Saadulla and Muhammed also showed that among 53 test isolates, the highest resistance was related to ceftriaxone (81.13%) and ciprofloxacin (79.24%), and the lowest resistance was related to colistin (5.66%) [27]. Finally, Ozkul and Hazırolan showed that among 44 bloodstream isolates, the highest resistance was related to trimethoprim-sulfamethoxazole (81.8%), while the lowest resistance was related to amikacin (68.2%) [28]. Accordingly, multiple studies have consistently demonstrated high antibiotic resistance among clinical isolates of *A*. *baumannii*.

The difference in antibiotic resistance rates in different studies could be explained by several factors [25]. For example, genetic variation in *A*. *baumannii* isolates, such as the presence of oxacillinase genes, affects resistance patterns [29]. Variation in biofilm formation, driven by genes such as *bap* and *bla*_PER−1_, also leads to different resistance levels [30]. Horizontal gene transfer enables the acquisition of new resistance genes; moreover, regional differences in antibiotic usage, particularly overuse of carbapenems and fluoroquinolones, create selective pressure, which affects antimicrobial resistance [31]. Additionally, hospital infection control practices impact the spread of resistant strains, further explaining the variation in resistance rates [32, 33].

According to these research results, 100% of the isolates developed biofilms. Based on the biofilm formation analysis results, the 53 examined *A*. *baumannii* isolates were divided into different categories: 67.92% were strong biofilm-producing isolates, 18.86% were moderate biofilm-producing isolates, and 11.32% were weak biofilm-producing isolates. In a study conducted by Khoshnood et al. on 50 *A*. *baumannii* isolates, the biofilm formation capacity was categorized as follows: 52% of the isolates were strong biofilm producers, 36% were moderate biofilm producers, and 12% were weak biofilm producers [34]. In another study conducted by Ghadiri, Doosti, and Shakhsi-Niaei, all *A*. *baumannii* isolates demonstrated biofilm formation capacity. Specifically, 12 isolates exhibited weak biofilm formation capacity, 28 isolates showed moderate biofilm formation capacity, and 50 isolates showed strong biofilm formation capacity [26]. Therefore, various studies have shown the high ability of *A*. *baumannii* isolates to form biofilms.

This study found a significant correlation between biofilm strength and antibiotic resistance in *A*. *baumannii* isolates, which aligns with other research findings. For example, Ghadiri, Doosti, and Shakhsi-Niaei observed that strong biofilm-forming isolates exhibited higher resistance to multiple antibiotics, which is similar to this study's results showing that strong biofilm producers were more resistant to antibiotics such as ampicillin/sulbactam, imipenem, and others [26]. Likewise, Khoshnood et al. demonstrated that the presence of biofilm-related genes was associated with increased antibiotic resistance, particularly among strong biofilm producers [34].

In this study, oxacillinase genes were identified among the isolates. Specifically, *bla*_OXA−23_, *bla*_OXA−24_, and *bla*_OXA−51_ genes were detected in 92.45%, 71.69%, and 100% of the isolates. Notably, none of the isolates carried the *bla*_OXA−58_ gene. Bardbari et al. reported that among 75 *A*. *baumannii* clinical isolates, the prevalence of *bla*_OXA−23_ and *bla*_OXA−24_ genes was 85.3% and 30.7%, respectively. In their study, similar to the present study findings, the *bla*_OXA−51_ gene was observed in all isolates, while the *bla*_OXA−58_ gene was not observed in any of the isolates [35]. In another study by Ozkul and Hazırolan, all *A*. *baumannii* isolates harbored the *bla*_OXA−51_ gene, and 63.6% were positive for the *bla*_OXA−23_ gene. None of the isolates were positive for *bla*_OXA−58_ and *bla*_OXA−24_ [28]. In another investigation by Vural et al., the *bla*_OXA−51_ gene was found in 100%, the *bla*_OXA−23_ gene in 96%, and the *bla*_OXA−24_ gene in 20.7% of *A*. *baumannii* isolates. None of the isolates harbored the *bla*_OXA−58_ gene [36]. Multiple studies have consistently reported the widespread presence of the *bla*_OXA−51_ gene in *A*. *baumannii* isolates, while the *bla*_OXA−58_ gene remains comparatively rare. The *bla*_OXA−51_ gene is intrinsic to *A*. *baumannii* and is chromosomally encoded in nearly all isolates, making it a reliable species-specific marker [37, 38]. In contrast, *bla*_OXA−58_ is an acquired gene, typically located on plasmids, and its transmission relies on horizontal gene transfer [39, 40]. The lower prevalence of *bla*_OXA−58_ is influenced by factors such as geographic distribution and local selective pressures, which shape the spread and persistence of this gene [37, 41].

Although *bla*_OXA−23_ and *bla*_OXA−24_ were more prevalent among strong biofilm producers in this study, no significant correlation was found between the presence of oxacillinase genes and biofilm strength (*p* > 0.05), suggesting that biofilm formation may be driven by other mechanisms rather than the direct influence of these resistance genes.

Based on the analysis of biofilm-encoding genes, *bap* and *bla*_PER−1_ genes were detected in 39 (73.58%) and 31 (58.49%) out of 53 *A*. *baumannii* isolates. In a study by Monfared et al., the *bap* gene was detected in 70.3% of *A*. *baumannii* isolates, while *bla*_−PER1_ was found in 54.2% [42]. Bardbari et al. reported that among *A*. *baumannii* clinical isolates, the prevalence of *bap* and *bla*_−PER1_ genes was 82.7% and 53.3%, respectively [35]. In another investigation by Saadulla and Muhammed, the frequencies of the biofilm-related genes *bap* and *bla*_PER−1_ among *A*. *baumannii* isolates were 79.24% and 9.43%, respectively [27].

This study offers a comprehensive analysis of biofilm formation and antibiotic resistance in *A*. *baumannii* clinical isolates by exploring the relationship between biofilm strength and antibiotic resistance using both phenotypic and molecular methods. The strength points of this study included the use of diverse clinical specimens and a multifaceted approach to understanding *A*. *baumannii* virulence. However, this study has some limitations, including the relatively small sample size, geographic focus, and lack of analysis of other biofilm-associated genes and clinical outcomes. Additionally, the lack of specific control tests, such as the ESBL test, restricted the study scope. Future research is suggested to consider larger sample sizes, broader gene ranges, and analysis of clinical outcomes to extend these findings.

## 5. Conclusion

This study demonstrated a high prevalence of antibiotic resistance among *A*. *baumannii* clinical isolates, all of which showed biofilm formation capacity. A significant correlation was found between biofilm strength and resistance to most antibiotics, underscoring the role of biofilm formation in the pathogen's ability to evade treatment. While all isolates carried the *bla*_OXA−51_ gene, none of them harbored the *bla*_OXA−58_ gene. Additionally, biofilm formation was significantly associated with the *bap* gene, highlighting its role in biofilm development, while the presence of oxacillinase genes showed no direct correlation with biofilm production.

These findings suggest that biofilm formation is a key factor contributing to the multidrug resistance of *A. baumannii* strains, making the treatment of associated infections more challenging. Targeting biofilm formation, particularly through the *bap* gene, could be a promising strategy for managing *A. baumannii* infections. Further research is needed to explore other biofilm-related mechanisms and develop more effective therapeutic approaches.

## Figures and Tables

**Table 1:** Sequences of the primers used in the study.

Genes	Forward and reverse primer sequences (5′ ⟶ 3′)	Amplicon size (bp)	Reference
*16s rRNA*	CATTATCACGGTAATTAGTG	208	[21]
	AGAGCACTGTGCACTTAAG		

*bla*_OXA−23_	GATCGGATTGGAGAACCAGA	501	[22]
	ATTTCTGACCGCATTTCCAT		

*bla*_OXA−24_	GGTTAGTTGGCCCCCTTAAA	246	[22]
	AGTTGAGCGAAAAGGGGATT		

*bla*_OXA−51_	TAATGCTTTGATCGGCCTTG	353	[22]
	TGGATTGCACTTCATCTTGC		

*bla*_OXA−58_	AAGTATTGGGGCTTGTGCTG	599	[22]
	CCCCTCTGCGCTCTACATAC		

*Bap*	TAGACGCAATGGATAACG	127	[23]
	TTAGAACCGATAACGATACC		

*bla*_PER−1_	GCAACTGCTGCAATACTCGG	900	[22]
	ATGTGCGACCACAGTACCAG		

**Table 2:** Antimicrobial susceptibility profiles of 53 *A*. *baumannii* isolates.

Antibiotic	Sensitive *N* (%)	Intermediate *N* (%)	Resistant *N* (%)
Ampicillin/sulbactam	18 (33.97)	—	35 (66.03)
Imipenem	4 (7.55)	—	49 (92.45)
Tobramycin	5 (9.43)	5 (9.43)	43 (81.13)
Amikacin	6 (11.33)	—	47 (88.67)
Ceftazidime	—	5 (9.44)	48 (90.56)
Trimethoprim/sulfamethoxazole	—	1 (1.89)	52 (98.11)
Ciprofloxacin	—	1 (1.89)	52 (98.11)

**Table 3:** Correlation between the specimen type and biofilm formation.

Sample type	Biofilm strength			*p* value
	Weak biofilm	Moderate biofilm	Strong biofilm	
Sputum *N* (%)	0	4 (23.52)	13 (76.47)	> 0.05
Blood *N* (%)	1 (8.33)	2 (16.66)	9 (75)	> 0.05
Wound *N* (%)	3 (30)	2 (20)	5 (50)	> 0.05
Urine *N* (%)	1 (10)	2 (20)	7 (70)	> 0.05
CSF *N* (%)	2 (50)	1 (25)	1 (25)	> 0.05

**Table 4:** Association between antimicrobial resistance and biofilm formation.

Antibiotics		Biofilm strength			*p* value
		Weak biofilm	Moderate biofilm	Strong biofilm	
Ampicillin/sulbactam	R	1	2	32	< 0.05
	I	0	0	0	
	S	5	9	4	

Imipenem	R	3	10	16	< 0.05
	I	0	0	0	
	S	3	1	0	

Tobramycin	R	1	8	34	< 0.05
	I	2	2	1	
	S	3	1	1	

Amikacin	R	3	9	35	< 0.05
	I	0	0	0	
	S	3	2	1	

Ceftazidime	R	4	9	35	< 0.05
	I	2	2	1	
	S	0	0	0	

Trimethoprim/sulfamethoxazole	R	5	11	36	< 0.05
	I	1	0	0	
	S	0	0	0	

Ciprofloxacin	R	6	11	35	> 0.05
	I	0	0	1	
	S	0	0	0	

Abbreviations: I = intermediate, R = resistant, S = susceptible.

**Table 5:** Association between oxacillinase genes and biofilm formation.

Antibiotic resistance genes	Number (%) of isolates	Biofilm strength *N* (%)			
		Weak (*N* = 6)	Moderate (*N* = 11)	Strong (*N* = 36)	*p* value
*bla*_OXA−23_	49 (92.45)	6 (12.24)	10 (20.4)	33 (67.34)	> 0.05
*bla*_OXA−24_	38 (71.69)	2 (5.26)	9 (23.68)	27 (71.05)	> 0.05
*bla*_OXA−51_	53 (100)	6 (11.32)	11 (20.75)	36 (67.92)	—

**Table 6:** Association between biofilm-encoding genes and biofilm formation.

Biofilm-encoding genes	Number (%) of isolates	Biofilm strength *N* (%)			
		Weak (*N* = 6)	Moderate (*N* = 11)	Strong (*N* = 36)	*p* value
*Bap*	39 (73.58)	1 (2.56)	4 (10.25)	34 (87.17)	< 0.05
*bla*_PER−1_	31 (58.49)	1 (3.22)	6 (19.35)	24 (77.41)	> 0.05

## Data Availability

The data that support the findings of this study are available from the corresponding author upon reasonable request.

## Supporting Information

Figure S1: Kirby–Bauer disc diffusion test. Figure S2: Biofilm formation assay by the crystal violet staining method. Figures S3, S4, and S5: Gel electrophoresis images of PCR products of oxacillinase and biofilm-formation genes.
