# Supplementary material for: Investigating the Link Between Biofilm Formation and Antibiotic Resistance in Clinical Isolates of Acinetobacter baumannii
Source: Int J Microbiol. 2025 Feb 12;2025:1009049. doi: 10.1155/ijm/1009049 (PMC11839262; doi:10.1155/ijm/1009049)
Supplement: Supporting Information — Additional supporting information can be found online in the Supporting Information section. [file 1009049.f1.docx]

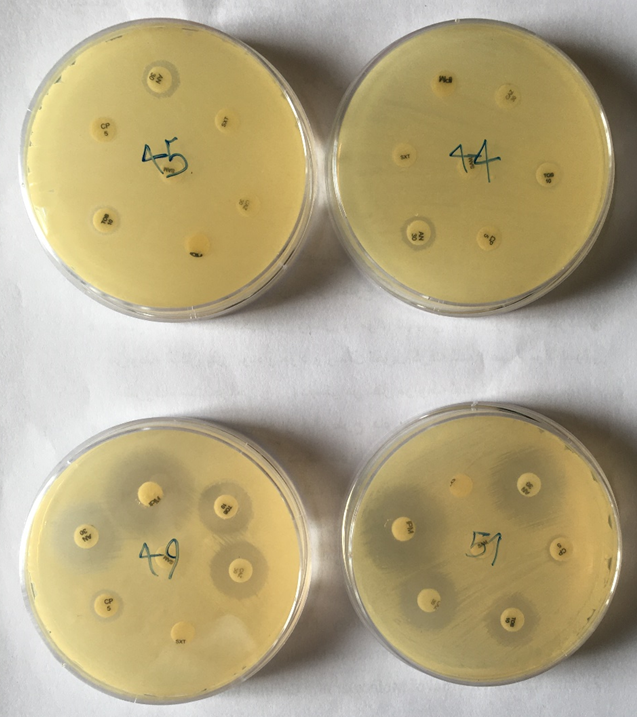


**Figure S1.** Kirby-Bauer disc diffusion test.


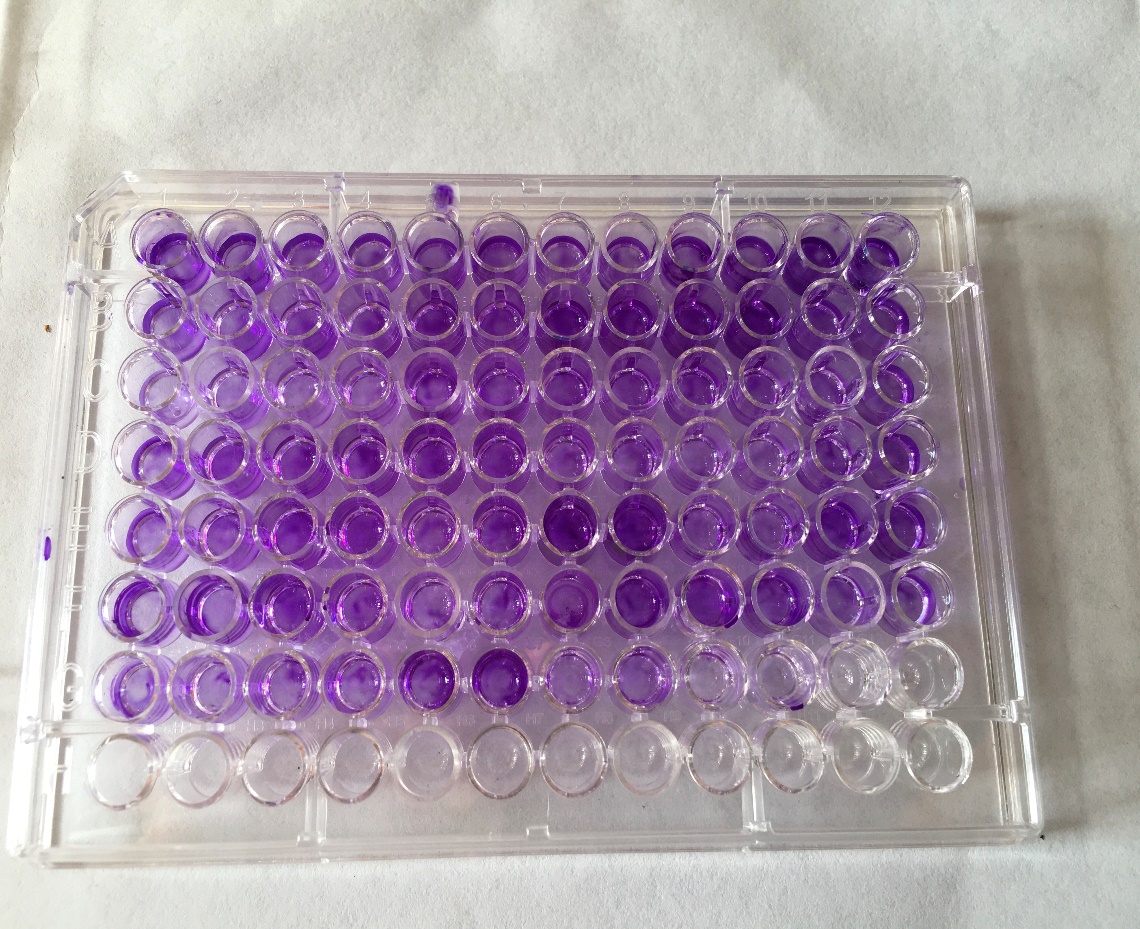


**Figure S2.** Biofilm formation assay by crystal violet staining method.


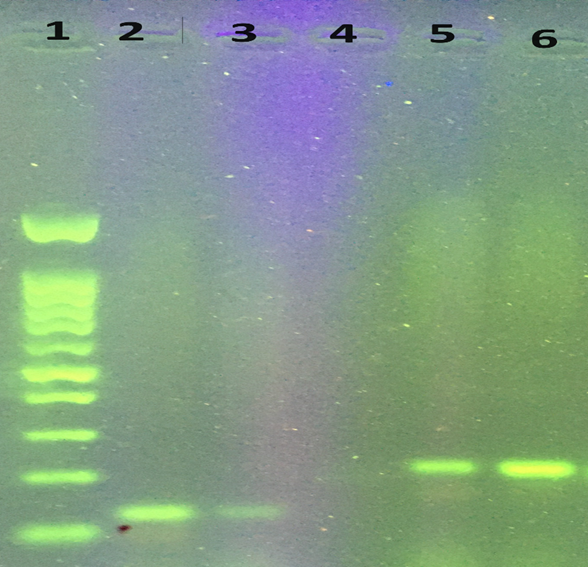


**Figure S3.** Well number 1: DNA Size Marker 100 bp, well numbers 2 and 3: *bap* positive (127 bp), well number 4: negative control, well number 5 and 6: *bla_OXA_*_-24_ positive (246 bp).


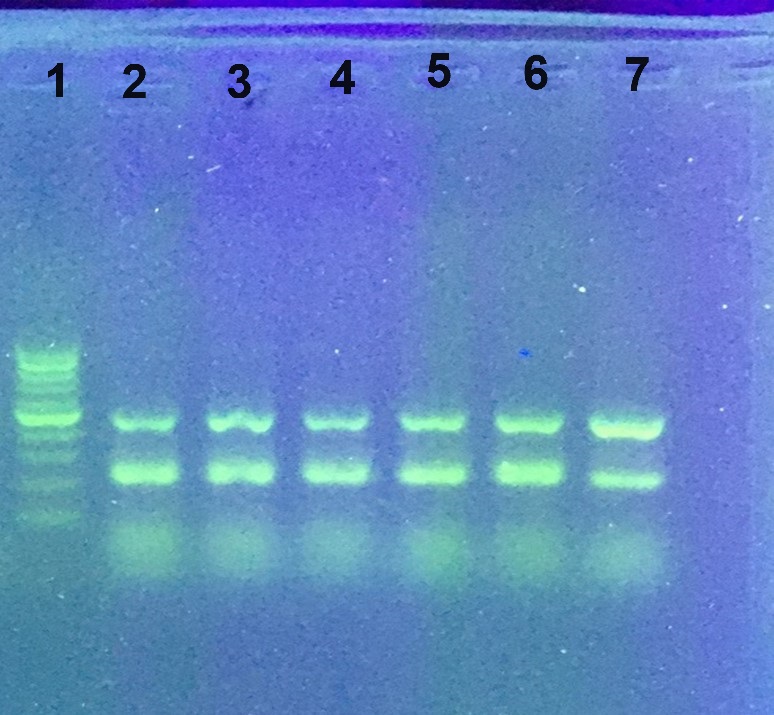


**Figure S4.** Well number 1: DNA Size Marker 100 bp, well numbers 2 to 7: *bla*_OXA-24_ (246 bp) and *bla*_OXA-23_ (501 bp) positive.


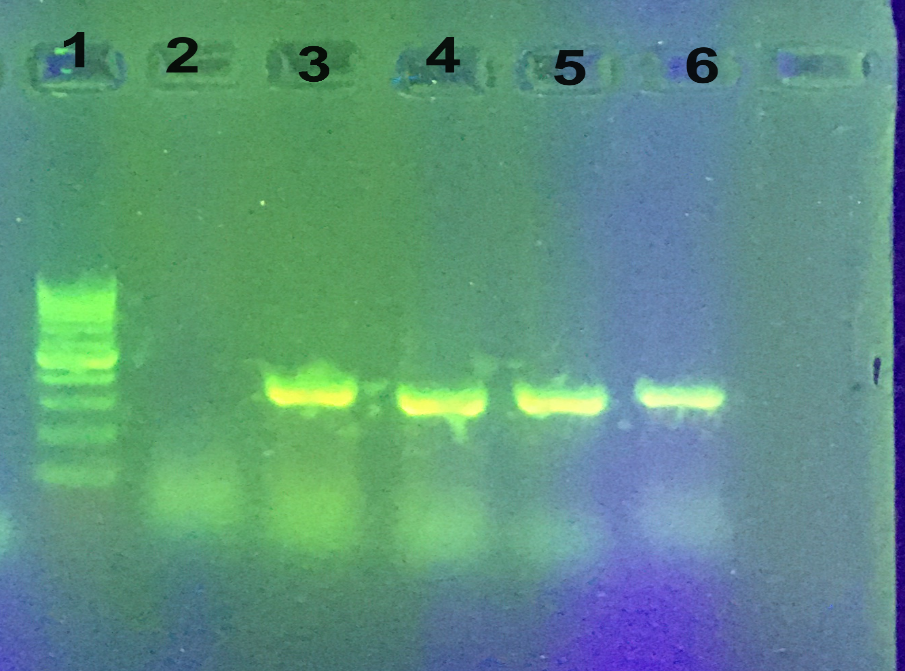


**Figure S5.** Well number 1: DNA Size Marker 100 bp, well number 2: negative control, well number 2 to 6: *bla*_OXA-51_ (353 bp) positive.
